# Dietary and physical activity behaviors among adults successful at weight loss maintenance

**DOI:** 10.1186/1479-5868-3-17

**Published:** 2006-07-19

**Authors:** Judy Kruger, Heidi Michels Blanck, Cathleen Gillespie

**Affiliations:** 1 Physical Activity and Health Branch, Division of Nutrition and Physical Activity, Centers for Disease Control and Prevention, Atlanta, Georgia, USA; 2 Chronic Disease Nutrition Branch, Division of Nutrition and Physical Activity, Centers for Disease Control and Prevention, Atlanta, Georgia, USA

## Abstract

**Background:**

There is limited population-based data on behavioral factors found to be important for successful weight loss maintenance among adults.

**Methods:**

Data from the 2004 Styles surveys, mailed to U.S. adults aged ≥18 years were used to examine the difference in selected weight loss strategies and attitudes among persons who reported successful weight loss attempts (lost weight and able to keep it off) and persons who were not successful (previous attempts to lose weight were unsuccessful or they could not keep the lost weight off). Behaviors examined included modification of diet, leisure-time and sports activities, and self-monitoring, and barriers to weight management.

**Results:**

Among adults who reported losing weight or trying to lose weight, 31.0% had been successful at both losing weight and maintenance after weight loss. Successful weight loss status differed by sex, age, and current weight status. Assessment of reported weight loss strategies, found that exercising ≥30 minutes/day and adding physical activity to daily life were significantly higher among successful versus unsuccessful weight losers. Individuals who were successful at weight loss and maintenance were less likely to use over-the-counter diet products than those who were unsuccessful at weight loss. Significantly more successful versus unsuccessful weight losers reported that on most days of the week they planned meals (35.9% vs. 24.9%), tracked calories (17.7% vs. 8.8%), tracked fat (16.4% vs. 6.6%), and measured food on plate (15.9% vs. 6.7%). Successful losers were also more likely to weigh themselves daily (20.3% vs. 11.0%). There were a significantly higher proportion of successful losers who reported lifting weights (19.0%) versus unsuccessful (10.9%). The odds of being a successful weight loser were 48%–76% lower for those reporting exercise weight control barriers were influencing factors (e.g., no time, too tired to exercise, no one to exercise with, too hard to maintain exercise routine) compared to those who reported little or no influence of exercise; similarly, the odds were 48–64% lower for those who found certain dietary barriers to be influential (e.g., eat away from home too often, diet/health food costs too much).

**Conclusion:**

Self-monitoring strategies such as weighing oneself, planning meals, tracking fat and calories, exercising 30 or more minutes daily, and/or adding physical activity to daily routine may be important in successful weight loss maintenance. Leisure-time activities such as lifting weights or cooking/baking for fun are common strategies reported by those who were successful weight losers.

## Background

Almost half of Americans are trying to lose weight [1]. Clinical trials have reported numerous short-term health benefits associated with weight loss including reductions in blood pressure and triglycerides, improvements in lipoprotein subfractions and insulin response, and better pulmonary function [2,3]. Long-term health benefits from weight loss include improved blood flow [4] and reduced cardiovascular disease mortality [5]. Unfortunately, many individuals who lose weight eventually regain most of the weight that was lost [6-8].

For successful weight loss, The National Heart, Lung, and Blood Institute *Guide to the Identification*, *Evaluation and Treatment of Overweight and Obesity in Adults *recommends using behavioral modification to reduce caloric intake and increase physical activity [9]. This may not be enough, however, as a person's readiness to lose weight may affect the chances of success [9]. In a review paper by Elfhag and Rossner (2005) they suggest that readiness and motivation failed to predict weight loss, however according to their review, successful weight maintenance is associated with immediate initial weight loss and goal attainment, an active lifestyle, self-monitoring weight-related behaviors, regular food intake patterns which include breakfast and healthier eating, and control over eating behavior [10]. Surveys of voluntary enrollees in the National Weight Control Registry, a cohort of 4000 adults age ≥18 years who have lost at least 13.6 kg (30 pounds) and kept it off at least 1 year, have found that the most common dietary strategies in this group to be restricting certain foods (mentioned by 87.5%), limiting quantities (44.0%), and counting calories (43.0%) for weight loss [8,11]. Another characteristic of registry members is high levels of physical activity. Energy expended by registry members represents approximately 1 hour of daily of moderate-intensity activity, such as brisk walking [8,11]. The most common activity in the cohort is walking (76%), and approximately 20% report weight lifting; 20% report cycling; and 18% aerobics. To date, this is the largest registry of people who have maintained their weight loss; however, there is limited information on population-based behavior strategies among persons who report being successful at weight loss.

The purpose of this descriptive paper is to examine differences in reported weight loss strategies, lifestyle activities, and perceived barriers to healthy weight among free-living adults who reported successful weight loss (lost weight and kept it off) and their counterparts who were not successful (lost weight but could not keep it off or were not able to lose weight). We describe the most common dietary practices, leisure-time physical activities, and sports, as well as self-monitoring behaviors (dietary measures and weighing oneself), and perceived barriers to weight loss, including time to exercise, cost of healthy foods, and social support.

## Methods

We examined data from Styles 2004, which is based on the results of three consumer mail panel surveys administered in two waves. The sampling and data collection for Styles 2004 were conducted by Synovate, Inc. The Synovate, Inc. consumer mail panel contains approximately 600,000 potential respondents. Respondents are recruited to join the mail panel through a 4-page questionnaire. In return for their participation, respondents were given small gifts (e.g., a 20-minute calling card) and were entered into a monetary sweepstakes. Content experts from several health agencies, including the Centers for Disease Control and Prevention, assisted with the development of questions. The initial wave – ConsumerStyles – was fielded May through June 2004. Stratified random sampling was used to generate a list of 10,000 potential respondents, who received the ConsumerStyles survey. The main sample (N = 5,500) was stratified (or balanced) on region, household income, population density, age, and household size to be representative of the U.S. population. A low-income/minority supplement (N = 1,500) was used to ensure adequate representation of these groups. Finally, a households-with-children supplement (N = 3,000) was used to ensure adequate numbers of potential respondents for a separate study of children called YouthStyles (part of the second wave). In 2004, a total of 6,207 people completed the ConsumerStyles survey, yielding a response rate of 62%.

Following the loss of 32 people from the ConsumerStyles panel during July-August 2004, the remaining 6,175 households that had completed the ConsumerStyles survey were mailed the HealthStyles survey. Responses were received from 4,345 of these households, for a response rate of 70%. Data on health and lifestyle used in the present analysis were mainly from the HealthStyles survey, while demographic characteristic were obtained from the ConsumerStyles.

### Definition of variables

#### Weight control behavior

Respondents self-identified their weight history experience. The lead-in to the questions on weight control history was: "Overall, what BEST describes your experience with your weight?" Respondents were asked to select one of the following: 1) I lost weight and have been able to keep it off, 2) I've lost weight but haven't been able to keep it off, 3) I've tried to lose weight but haven't been successful, 4) I've maintained my weight with conscious effort, 5) I've maintained my weight without effort, 6) I've gained weight and haven't tried to lose it, and 7) I pay no attention to my weight. Participants who reported that they lost weight and had been able to keep it off were defined as "successful weight losers", those who reported they had lost weight, but were unable to keep it off or had tried to lose weight but had not been successful were defined as "unsuccessful weight losers".

#### Weight control strategies

The 2004 Styles survey asked respondents about strategies to aid with losing weight or maintaining weight loss: "Below are some strategies people use to lose or maintain their weight. In the past 12 months, which of the following have you tried?" There were 18 strategies: 1) Reduce the amount of food you eat, 2) Exercise an average of at least 30 minutes per day, 3) Eat more fruits and vegetables, 4) Eat reduced calorie products, 5) Reduce high carbohydrate foods like bread or potatoes, 6) Eat smaller portion sizes, 7) Cut out sweetened beverages, 8) Eat reduced-carbohydrate food products, 9) Eat reduced-fat-products, 10) Count calories, 11) Reduce sedentary activities, 12) Consume over-the-counter diet products, 13) Reduce the amount of food prepared away from home, 14) Consume meal replacement products, 15) Incorporate physical activity into daily routines, 16) Go to formal weight loss program, 17) Keep a food diary, or 18) Use Internet web-site with individualized diet program. Respondents were also asked to report on their weekly weight control practices: "Which of the following, if any, do you do most days of the week?" and were asked to select all that applied from a list of 6 items. These items included: 1) Track how many calories you eat, 2) Track how many carbohydrates you eat, 3) Track how many grams of fat you eat, 4) Plan your meals and snacks throughout the day, 5) Think about the amount of food you put on your plate, and 6) Measure the amount of food you put on your plate. Respondents who did not select any of the listed activities were classified as "not specified". An additional question on the frequency of weighing oneself was asked in a separate question, "How frequently do you weigh yourself?" Respondents could select every day, every week, every month, every couple of months, about once a year or less, or never.

#### Physical activity/sports and leisure-time activity

Respondents were also asked to report all physical activities/sports they engage in from a list of 18 specific activities: "Which of the following physical activities/sports do you participate in regularly?" These items included baseball/softball, basketball, jogging/running, walking, biking, bowling, golf, exercise class, lifting weights, yoga/meditation, karate/martial arts, swimming, hiking, hunting/fishing, camping, skiing/snowboarding, tennis, and volleyball. In addition, respondents were asked to select leisure-time activities that they enjoyed doing regularly from a list of 29 possible activities. For descriptive purposes, we combined various items considered similar in energy expenditure (e.g., playing computer games and playing video games). Respondents who did not select any of the activities were classified as "not specified".

#### Barriers to losing weight or maintaining weight loss

Respondents were asked about 9 possible barriers to weight control: "The following is a list of possible reasons that keep people from losing weight or maintaining a healthy weight." For each item, respondents were asked to characterize its influence using a 7-point Likert scale (1 = no or little influence; 7 = influences a lot). The items were as follows: 1) I eat away from home too often, 2) I'm often too tired to exercise, 3) I like to eat junk food/have a sweet tooth, 4) I don't have time to exercise, 5) I don't really pay attention to what I'm eating, 6) I don't have anyone to exercise with me, 7) Diet/healthy foods are not as filling/still feel hungry, 8) It is too hard to stick with an exercise routine, and 9) Diet/healthy foods cost too much. For regression analysis, responses 1–3 were combined to create "no influence", 4 represented neutral and 5–7 were combined to represented "influence."

### Statistical analysis

From the 4,345 respondents to HealthStyles, we chose as our analytic sample to the 2,124 participants who were classified as successful weight losers (n = 587, 14.4%) or unsuccessful weight losers (n = 1,537, 32.1%). We excluded respondents who had maintained weight with or without effort (n = 1,363, 32.9%), gained weight and had not tried to lose it (n = 355, 8.5%), paid no attention to their weight (n = 304, 7.6%) or were missing data on weight loss or maintenance (n = 199, 4.7%). Participants with missing height or weight (n = 77) or those who reported extreme height or weight values (outside the 1^st^–99^th ^percentile in the 1999–2002 National Health and Nutrition Examination Survey) (n = 44) were also excluded. We also excluded female respondents who stated that they were currently pregnant or who did not respond to the question on pregnancy (n = 52). After exclusions the final sample numbered 1,958 (some participants met one or more exclusion criteria).

We compared successful and unsuccessful weight losers by their demographic characteristics, weight-loss strategies, self-monitoring behaviors, and activities also compared them by nine barriers described. Because of the multiple testing involved, we selected p = 0.01 as our level of significance for the chi-square test to reduce the type II error probability. We used multivariable logistic regression to calculate odds ratios (ORs) with 95% confidence intervals (CIs) for successful weight loss (versus unsuccessful) in a model that included sex, age, race, education, annual household income and body mass index (BMI; calculated as weight in kilograms divided by height in meters squared). We also used multivariable logistic regression models that adjusted for sex, age, race, education, annual household income, and BMI to assess the relation between being a successful weight loser and specific barriers to losing or maintaining weight. Missing data because of no response were excluded from the individual comparisons. All analyses were conducted using SAS-callable (version 9.1, Cary, NC) SUDAAN (version 9.0 Research Triangle Institute, Research Triangle Park, NC) to account for the complex sampling design and weighting procedure.

## Results

In our final analytic sample of successful and unsuccessful weight losers, one-third (30.96%) were successful at losing weight and keeping their weight off. The chances of being in the successful group were lower for women (adjusted OR = 0.63, 95%CI = 0.48-0.81) than men (the referent) (Table 1). Lower odds of being a successful weight loser were observed for those aged 30–44 years (OR = 0.51, 95%CI = 0.30-0.89) than those aged 18–29 years (referent). The odds of being a successful weight loser did not differ by race/ethnicity, education, or household income. The odds of being in the successful group were far lower for those persons with a BMI ≥ 35.0 (OR = 0.12, 95%CI = 0.07-0.19) than in the reference group (BMI <25.0).

**Table 1:** Prevalence of successful and unsuccessful weight loss and adjusted odds of being successful by demographic characteristics – Styles Survey 2004.

	**Sample size**	**Successful**		**Unsuccessful**			
	N^a^	%	(se)^b^	%	(se)	aOR^c^	(95% CI)
Sex	1,958	30.96	(1.35)	69.04	(1.35)		
Male	745	35.78	(2.20)	64.22	(2.20)	1.00	
Female	1,213	27.36	(1.67)	72.64	(1.67)	0.63	(0.48–0.81)
Age							
18–29	132	39.72	(5.66)	60.28	(5.66)	1.00	
30–44	713	25.62	(1.82)	74.38	(1.82)	0.51	(0.30–0.89)
45–64	811	25.83	(1.67)	74.17	(1.67)	0.58	(0.34–1.00)
65+	302	44.05	(3.04)	55.95	(3.04)	1.21	(0.69–2.12)
Race/ethnicity							
White	1,404	29.65	(1.51)	70.35	(1.51)	1.00	
Black	204	32.99	(4.06)	67.01	(4.06)	1.50	(0.96–2.32)
Hispanic	234	34.05	(4.35)	65.95	(4.35)	1.20	(0.81–1.79)
Other^d^	116	37.03	(6.90)	62.97	(6.90)	1.88	(0.51–1.53)
Education							
College grad	571	34.03	(2.47)	65.97	(2.47)	1.00	
Some College	716	27.34	(2.07)	72.66	(2.07)	0.78	(0.55–1.09)
<=High school	599	29.35	(2.27)	70.65	(2.27)	0.84	(0.58–1.24)
missing	72	43.71	(7.88)	56.29	(7.88)	1.28	(0.63–2.61)
Annual household income							
>60 K	744	29.00	(1.81)	71.00	(1.81)	1.00	
25–60 K	647	30.89	(2.41)	69.11	(2.41)	1.23	(0.90–1.68)
<25 K	567	33.79	(2.89)	66.21	(2.89)	1.15	(0.79–1.67)
Body mass index							
<25.0	383	58.55	(2.99)	41.45	(2.99)	1.00	
25.0<=<30.0	655	32.27	(2.39)	67.73	(2.39)	0.31	(0.22–0.42)
30.0<=<35.0	502	20.18	(2.13)	79.82	(2.13)	0.16	(0.11–0.24)
>35.0	418	14.70	(2.63)	85.30	(2.63)	0.12	(0.07–0.19)

^a ^Unweighted sample size.

^b ^Standard error.

^c ^Odds ratio adjusted for sex, age, race/ethnicity, education, income, and BMI. CI indicates confidence interval.

^d ^Includes American Indian, Alaska Native, Asian, Native Hawaiian, and Other Pacific Islander.

The five most common weight-control behaviors did not differ significantly (p > 0.005) between successful and unsuccessful weight losers: reduced amount of food consumed (79.60%; 82.39%), more fruits and vegetables consumed (71.37%; 65.91%), smaller portions (64.57%; 64.98%), fewer fatty foods (60.13%; 57.66%), and no sweetened beverages (56.50%; 52.57%) (Table 2). In contrast, a significantly higher proportion of successful weight losers reported exercising 30 or more minutes daily (the sixth most common strategy (46.91% versus 37.54%) and adding physical activity to their daily routine (46.70% versus 34.88%; this was the seventh most common strategy). Alternately, a significantly lower proportion of successful weight losers reported using over-the-counter diet products (the seventeenth most common strategy (10.36% versus 15.89%).

**Table 2:** Most common weight-loss strategies reported among successful losers and unsuccessful weight losers – Styles Survey, 2004

		**Successful**		**Unsuccessful**		
		(n = 543)		(n = 1,415)		χ^2^
	n	%	(se)^a^	%	(se)^a^	p-value
**Strategy:**						
Reduced amount of food	1,602	79.60	(2.21)	82.39	(1.19)	0.268
More fruits & vegetables	1,325	71.37	(2.25)	65.91	(1.53)	0.049
Smaller portions	1,271	64.57	(2.69)	63.98	(1.51)	0.849
Fewer fatty foods	1,134	60.13	(2.68)	57.66	(1.55)	0.425
No sweetened beverages	1,001	56.50	(2.67)	52.57	(1.56)	0.207
Exercise ≥30 min/day	780	46.91	(2.73)	37.54	(1.52)	0.003*
Add physical activity to daily routine	735	46.70	(2.75)	34.88	(1.48)	<0.001*
Reduce high-carbohydrate foods	882	40.45	(2.58)	45.36	(1.55)	0.108
Eat reduced-fat products	696	39.61	(2.70)	35.10	(1.51)	0.147
Reduced-calorie products	767	38.50	(2.61)	40.35	(1.56)	0.542
Reduce food prepared away from home	702	36.36	(2.62)	37.84	(1.58)	0.628
Eat reduced-carbohydrate food products	521	26.00	(2.23)	26.67	(1.44)	0.800
Reduce sedentary activity	379	21.96	(2.12)	16.97	(1.17)	0.039
Count calories	348	19.74	(2.09)	18.34	(1.32)	0.572
Food diary	251	12.97	(1.71)	13.05	(1.13)	0.965
Meal-replacement products	284	10.82	(1.66)	15.48	(1.08)	0.019
Over-the-counter diet products	273	10.36	(1.81)	15.89	(1.13)	0.010*
Formal weight-loss program	158	7.37	(1.48)	8.47	(0.81)	0.514
Web-site with individualized diet plan	73	2.60	(0.64)	5.33	(0.92)	0.016
Not specified	25	4.1	(0.90)	2.4	(0.44)	0.086

^a ^Standard error.

*Significant difference between successful and unsuccessful (p < 0.01).

Analysis of self-monitoring behaviors (conducted most days of the week) showed numerous significant differences by weight loss status (Table 3). A significantly higher proportion of successful weight losers reported planning meals (35.90% versus 24.88%) tracking calories (17.73% versus 8.84%), and tracking fat (16.40% versus 6.57%). Similarly, a higher proportion of successful losers reported measuring the amount of food on their plate (15.89% versus 6.73%). A higher proportion of successful weight losers weighed themselves every day (20.30%) compared to those who were unsuccessful (11.00%) at losing weight.

**Table 3:** Self-monitoring behaviors (dietary and body weighing) done on most days of the week among successful and unsuccessful weight losers – Styles Survey, 2004

		**Successful**		**Unsuccessful**		
		(n = 543)		(n = 1,415)		χ^2^
**Behavior:**	n	%	(se)^a^	%	(se)^a^	p-value
Monitor amount of food on plate	1,169	63.22	(2.70)	56.80	(1.57)	0.039
Plan meals	559	35.90	(2.61)	24.88	(1.35)	0.000*
Track carbohydrates	293	18.40	(1.94)	13.51	(1.05)	0.026
Track calories	226	17.73	(1.99)	8.84	(0.80)	<0.001*
Track fat	181	16.40	(1.97)	6.57	(0.73)	<0.001*
Measure food on plate	187	15.89	(1.89)	6.73	(0.76)	<0.001*
Not specified	399	14.06	(2.27)	24.61	(1.44)	0.001*
Weigh oneself (frequency)						
Daily	270	20.30	(2.03)	11.00	(0.91)	
Less often	1,674	79.70	(2.03)	89.00	(0.91)	<0.001

^a ^Standard error.

*Significant difference between successful and unsuccessful (p < 0.01).

The item about the frequency of weighing oneself was asked in a separate question.

Among both successful and unsuccessful weight losers, walking was the most common physical activity/sports (61.51% versus 56.41% difference not significant) (Table 4). The only significant difference was for lifting weights (19.01% of successful, 10.86% of unsuccessful, p = 0.002). Both successful and unsuccessful weight losers engaged in a variety of leisure-time activities, but, only cooking/baking for fun was more common among those who were successful (45.59% versus 36.48%) (p = 0.004) (Table 5).

**Table 4:** Physical activities/sports reported by successful losers and unsuccessful weight losers – Styles Survey, 2004

		**Successful**		**Unsuccessful**		
		(n = 543)		(n = 1,415)		χ^2^
**Activity:**	n	%	(se)^a^	%	(se)^a^	p-value
Walking	1,152	61.51	(2.65)	56.41	(1.57)	0.100
Lifting weights	229	19.01	(2.31)	10.86	(1.11)	0.002*
Biking	219	15.95	(2.35)	10.51	(0.95)	0.035
Hunting/fishing	231	15.05	(2.17)	11.54	(1.10)	0.152
Exercise class	209	13.77	(2.07)	9.35	(0.82)	0.049
Camping (overnight)	262	13.17	(1.71)	14.09	(1.26)	0.665
Swimming	240	11.93	(1.77)	12.83	(1.07)	0.664
Jogging/running	136	9.86	(1.57)	6.71	(0.79)	0.073
Hiking	143	8.07	(1.45)	8.00	(0.99)	0.968
Basketball	81	7.68	(1.91)	3.81	(0.62)	0.057
Golf	122	7.52	(1.24)	6.83	(0.83)	0.647
Bowling	126	7.34	(1.73)	7.09	(0.81)	0.895
Not specified	95	15.0	(1.79)	22.7	(1.31)	0.001
Yoga/meditation	83	5.52	(1.13)	4.07	(0.66)	0.268
Baseball/softball	79	5.43	(1.20)	3.97	(0.62)	0.278
Tennis	42	3.61	(1.04)	2.17	(0.51)	0.214
Volleyball	51	2.83	(1.01)	3.23	(0.65)	0.742
Skiing/snowboarding	38	2.21	(0.61)	2.16	(0.52)	0.951
Karate/martial arts^b^	18	1.65	(0.59)	0.42	(0.15)	0.045

*Significant difference between successful and unsuccessful significant (p < 0.01).

^a ^Standard error.

^b ^Estimates are unstable by National Center for Health Statistics standards (se [standard error]/estimate>0.3).

**Table 5:** Leisure-time activities reported by successful losers and unsuccessful weight losers – Styles Survey, 2004

		**Successful**		**Unsuccessful**		
		(n = 543)		(n = 1,415)		χ^2^
**Activity:**	n	%	(se)^a^	%	(se)^a^	p-value
Listening to music	1,609	85.43	(1.60)	82.79	(1.09)	0.178
Reading	1,162	60.67	(2.72)	57.17	(1.57)	0.264
Arts/crafts/collecting	1,041	50.67	(2.74)	53.08	(1.56)	0.446
Going out to or renting movies	946	46.66	(2.79)	51.05	(1.57)	0.165
Playing cards/board games	926	45.70	(2.74)	47.09	(1.57)	0.659
Cooking/baking for fun	772	45.59	(2.74)	36.48	(1.49)	0.004*
Playing computer/video games	851	45.56	(2.81)	45.19	(1.58)	0.907
Shopping for fun	829	44.04	(2.72)	40.55	(1.51)	0.264
DIY home projects/parties	815	43.39	(2.72)	41.10	(1.56)	0.466
Gardening	840	42.78	(2.61)	38.65	(1.50)	0.167
Picnics/parks	615	32.75	(2.53)	30.66	(1.52)	0.478
Going to beach/lake	604	32.29	(2.61)	32.05	(1.51)	0.937
Gambling/casino/lottery tickets	663	31.67	(2.48)	37.67	(1.58)	0.043
Zoo/theme/water parks	584	30.35	(2.60)	31.02	(1.52)	0.826
Religious activities	585	28.92	(2.47)	28.12	(1.36)	0.777
Going to theater/symphony	380	22.56	(2.31)	19.22	(1.18)	0.199
Attending sports events	437	22.38	(2.28)	23.55	(1.37)	0.659
Going to nightclubs/bars	201	15.42	(2.53)	11.70	(1.16)	0.188
Book club	216	10.92	(1.55)	11.12	(1.04)	0.915
Adult education classes	148	8.28	(1.30)	6.49	(0.68)	0.222
Not specified	4	0.7	(0.36)	0.8	(0.29)	0.822

^a ^Standard error.

*Significant difference between successful and unsuccessful significant (p < 0.01).

The following groups on the questionnaire were collapsed: playing cards and playing board games; playing computer games and playing video games; arts & crafts, collecting (dolls, cards), sewing/needlework, photography, and woodworking; gambling/casino and lottery tickets/scratch off; zoo and theme parks.

After adjustment for covariates, the odds of being a successful weight loser were 48%–76% lower for those reporting that exercise aspects were influencing factors (i.e., no time to exercise, too tired to exercise, no one to exercise with, too hard to maintain exercise routine) compared to those who reported little or no influence of exercise as a weight control barrier (Table 6). The odds of being a successful weight loser were 48–64% lower for those who were influenced by dietary barriers to weight control (eat away from home too often, like to eat junk food, don't pay attention to diet, diet/health foods not satisfying, diet/health food costs too much) compared to those who reported little or no influence of diet. Respondents who had neutral feelings about the barriers to losing or maintaining healthy weight had lower odds of success than those who thought these barriers were not influential.

**Table 6:** Prevalence of successful and unsuccessful weight loss by the influence of various barriers and odds of successful weight loss by barrier-Styles Survey, 2004

		**Successful**		**Unsuccessful**			
		(n = 543)		(n = 1,415)			
	n	%	(se)^a^	%	(se)^a^	OR^b^	95% CI
**Barrier**^**c**^:							
Too tired to exercise							
No influence	551	48.45	(2.97)	20.95	(1.21)	1.00	
Neutral	285	16.68	(2.49)	14.33	(1.05)	0.49	(0.33, 0.72)
Influence	1078	34.87	(2.60)	64.72	(1.46)	0.24	(0.19, 0.32)
No time to exercise							
No influence	773	55.56	(2.79)	35.17	(1.50)	1.00	
Neutral	374	16.66	(2.33)	20.35	(1.31)	0.46	(0.33, 0.65)
Influence	765	27.78	(2.46)	44.48	(1.58)	0.41	(0.31, 0.56)
Too hard to maintain exercise routine							
No influence	636	51.71	(2.80)	24.99	(1.30)	1.00	
Neutral	353	18.72	(2.39)	18.62	(1.21)	0.42	(0.31, 0.59)
Influence	929	29.58	(2.58)	56.40	(1.55)	0.26	(0.20, 0.35)
No one to exercise with							
No influence	985	62.24	(2.76)	46.46	(1.57)	1.00	
Neutral	292	12.21	(1.51)	15.29	(1.17)	0.67	(0.48, 0.95)
Influence	627	25.55	(2.67)	38.25	(1.60)	0.52	(0.39, 0.70)
Eat away from home too often							
No influence	973	61.50	(2.89)	42.42	(1.54)	1.00	
Neutral	292	12.71	(2.18)	17.18	(1.20)	0.56	(0.37, 0.84)
Influence	656	25.80	(2.69)	40.40	(1.59)	0.36	(0.27, 0.49)
Diet/health foods not satisfying							
No influence	873	59.54	(2.83)	41.10	(1.55)	1.00	
Neutral	398	22.09	(2.14)	22.09	(1.33)	0.58	(0.40, 0.83)
Influence	637	23.87	(2.61)	36.82	(1.54)	0.44	(0.33, 0.59)
Don't pay attention to diet							
No influence	853	61.17	(2.60)	40.18	(1.54)	1.00	
Neutral	455	20.17	(2.17)	25.22	(1.38)	0.59	(0.43, 0.82)
Influence	593	18.66	(1.80)	34.60	(1.55)	0.37	(0.28, 0.49)
Like to eat junk food							
No influence	754	53.46	(2.81)	33.16	(1.45)	1.00	
Neutral	345	17.08	(2.26)	19.29	(1.28)	0.50	(0.35, 0.70)
Influence	824	29.46	(2.65)	47.55	(1.58)	0.38	(0.28, 0.50)
Diet/health food costs too much							
No influence	756	49.53	(2.78)	34.96	(1.46)	1.00	
Neutral	273	12.01	(1.88)	16.15	(1.24)	0.61	(0.41, 0.92)
Influence	888	38.46	(2.87)	48.88	(1.59)	0.52	(0.39, 0.68)

^a ^Standard error.

^b ^Includes participants with complete data for each barrier.

^a ^Odds ratio adjusted for sex, age, race, education, income, and BMI. CI indicates confidence interval.

Each predictor variable is examined separately.

## Discussion

Among 1,958 HealthStyles respondents who had lost weight or tried to lose weight, almost one-third had been succeeded in losing weight and keeping it off. Overall, successful weight losers and maintainers were more likely to engage in physical activity for at least 30 minutes per day, or to add physical activity to their daily routine than those unsuccessful at weight loss and maintenance. Finding suggests that individuals who were successful at weight loss and maintenance had higher odds of taking part in physical activity on most days of the week than those who were unsuccessful at weight loss. Interestingly, individuals who were successful at weight loss and maintenance had lower odds of using over-the-counter diet products than those who were unsuccessful at weight loss. Similarly, self-monitoring behaviors such as weighing oneself daily were also reported more often by successful weight losers than unsuccessful weight losers. There is earlier evidence that people who weigh themselves at least weekly are more likely to lose weight and avoid regaining it than those who weigh themselves inconsistently [12,13]. In addition, self-monitoring of body weight was found to be an important factor among members of the National Weight Control Registry, a group of successful weight losers [8]. In a comprehensive review by Teixeira and colleagues (2005), a self-motivated cognitive style was found to predictor compliance to a multitude of behaviors necessary for successful weight management [14].

Questions on a wide range of weight-control strategies (e.g., counting calories, reducing amount of food, eating fewer fatty foods, consuming reduced-fat products) revealed no differences by success at losing weight, but these questions referred to having tried something in the past 12 months. When the focus was weight-control practices that were followed most days of the week, the results were significantly different, as planning meals, tracking calories, tracking fat, and measuring food were all more common in successful weight losers than unsuccessful weight losers. Others have found similar results [8]. Gorin and colleagues [15] found that consistency in dieting through the week and year was better for maintaining weight loss than simply restricting dieting to weekdays. Although it makes intuitive sense (and is consistent with our study) that tracking one's behavior may lead to better health practices, with the exception of food diaries, there is a lack of research to support this hypothesis.

Although not a specific physical activity recommendation for weight loss, weight training may, through various mechanisms increase the overall expenditure of energy, and thereby aid in weight control [16,17]. Much more needs to be known about how weight training may aid in weight maintenance, as it has been reported by 20% of members of the National Weight Control Registry [9,18]. In our study, weight lifting was almost twice as common among successful weight losers, and we also found that men were more likely than women to be successful at losing weight. One might conclude that a greater predilection for weight lifting was part of the reason for the men's greater success in losing weight, or perhaps, weight lifting was for many participants a way of training for specific sports and thus, possibly a marker for a physically active lifestyle.

The odds of being a successful weight loser were lower for those who reported being influenced by dietary weight control barriers (e.g., eat away from home too often, diet/health foods not satisfying, diet/health food costs too much) compared to those who reported little to no influence of dietary barriers on weight control. This suggests that issues of taste, cost and convenience may need to be included in initiatives aimed at helping individuals lose or maintain weight. Our finding that cooking or baking for fun was more common among those successful at weight loss was by no means unexpected but perhaps not as predictable. Perhaps those who enjoy cooking and baking have meals at home more often; meals that one would expect to be healthier than those in fast-food or full-service restaurants. The nutritional quality of food prepared at home has been found to be superior to foods prepared away from home, with the latter being higher in total calories, fat, and sodium and lower in fiber [19].

It was also not surprising that barriers to exercise (including being too tired, having no time or no one with whom to exercise, finding it too hard to maintain an exercise routine) were associated with being unsuccessful at losing weight. Thus, both time pressure and lack of social support seem important. Elfhag and Rossner (2005) found that social support, better coping strategies and the ability to handle life stress were factors associated with successful weight maintenance [10]. The Task Force on Community Preventive Services [20] suggests that environmental barriers and lack of social support influence exercise behavior. Increasing convenient opportunities to engage in lifestyle physical activity (e.g., making small changes to the built environment, such as sidewalks, parks, and trails) may motivate some sedentary people to change their behavior.

The findings of this analysis are subject to several limitations. First, data from the HealthStyles survey are cross-sectional, and thus causality cannot be determined. Second, participants are not randomly drawn from the US population, but results of the HealthStyles survey have been shown to be comparable to those obtained through the Behavioral Risk Factor Surveillance System (BRFSS), which does use a probability sampling technique [21,22]. Specifically, nine items on the HealthStyles survey were comparable to items on the BRFSS survey from 1995 to 2001, yielding 34 same-year data pairs where the two surveys could be compared directly [21]. The average difference for the 34 pairs of percentages was 2.4 percentage points, and the correlation between the 34 pairs was r = 0.99 [22]. A third concern is that our primary survey questions have not been tested for validity or reliability. Fourth, the questionnaire did not include questions as to how much weight was lost, which limits the definition of a successful weight loss maintainer. While there is no consensus in the literature about how to define weight maintenance, it is important to consider defining long-term maintenance in terms of the amount of weight change and time frame of the occurrence [23]. Finally, the questionnaire provided limited details about the intensity or duration of most of the physical activities/sports, and it lacked an objective measure of how much weight was lost and kept off. The strengths of this study include the fact that it was drawn from a sample of American households and that the survey questions allowed for the categorization of an assortment of dietary variables, leisure-time activities, and physical activities or sports that might be associated with successful and unsuccessful weight loss.

## Conclusion

Because maintenance after weight loss can be difficult, it is important to identify factors that facilitate success. Self-monitoring strategies such as weighing oneself, planning meals, tracking fat and calories, exercising 30 or more minutes daily, and adding physical activity to the daily routine may be important in maintaining weight loss. The results also indicate that successful weight losers were more likely to report lifting weights or cooking/baking for fun compared to unsuccessful weight losers.

## Abbreviations

Standard error, SE

## Competing interests

The author(s) declare that they have no competing interests.

## Authors' contributions

JK carried out the plan for analysis, interpretation of the data,, and writing of the manuscript. HMB developed the survey questions and aided in the plan for analysis, interpreting the data, and writing the manuscript; CG analyzed the statistics and aided in interpreting the results and in editing the manuscript. All three authors read and approved the final manuscript.
